# Primary Ciliary Dyskinesia: A Clinical Review

**DOI:** 10.3390/cells13110974

**Published:** 2024-06-04

**Authors:** Katherine A. Despotes, Maimoona A. Zariwala, Stephanie D. Davis, Thomas W. Ferkol

**Affiliations:** 1 Department of Pediatrics, UNC School of Medicine, The University of North Carolina at Chapel Hill, Chapel Hill, NC 27599, USA; 2 Marsico Lung Institute, UNC School of Medicine, The University of North Carolina at Chapel Hill, Chapel Hill, NC 27599, USA

**Keywords:** primary ciliary dyskinesia, cilia, motile ciliopathy, bronchiectasis, genotype, phenotype

## Abstract

Primary ciliary dyskinesia (PCD) is a rare, genetically heterogeneous, motile ciliopathy, characterized by neonatal respiratory distress, recurrent upper and lower respiratory tract infections, subfertility, and laterality defects. Diagnosis relies on a combination of tests for confirmation, including nasal nitric oxide (nNO) measurements, high-speed videomicroscopy analysis (HSVMA), immunofluorescent staining, axonemal ultrastructure analysis via transmission electron microscopy (TEM), and genetic testing. Notably, there is no single gold standard confirmatory or exclusionary test. Currently, 54 causative genes involved in cilia assembly, structure, and function have been linked to PCD; this rare disease has a spectrum of clinical manifestations and emerging genotype–phenotype relationships. In this review, we provide an overview of the structure and function of motile cilia, the emerging genetics and pathophysiology of this rare disease, as well as clinical features associated with motile ciliopathies, novel diagnostic tools, and updates on genotype–phenotype relationships in PCD.

## 1. Introduction

Primary ciliary dyskinesia (PCD) is a genetically heterogeneous, motile ciliopathy characterized by ineffective ciliary movement leading to recurrent upper and lower respiratory tract infections, neonatal respiratory distress, subfertility, and laterality defects. “Primary” indicates a genetic or inherited cause of ciliary dysfunction, distinct from “secondary” or acquired causes of ciliary dysfunction, typically related to epithelial injury in the airway from insults including infections and pollution [1]. Population estimates of the prevalence of PCD have ranged from 1:10,000 to 1:30,000, though these values are likely underestimates due to under-recognition and difficulty in diagnosing this condition [2,3,4,5]. More recently, based on the frequency of 29 known genes and pathogenic variants, the calculated prevalence may be closer to 1:7500 worldwide [6]. 

With advances in cilia genetic testing coupled with the improved understanding of phenotype, a growing number of genes have been implicated in PCD. The severity of symptoms and clinical phenotypes vary, and genotype–phenotype relationships have recently emerged. In this review, we provide an overview of the structure and function of motile cilia, the emerging genetics and pathophysiology of this rare disease, as well as clinical features associated with motile ciliopathies, novel diagnostic tools, and updates on genotype–phenotype relationships in PCD. 

## 2. Cilia and Their Role in Health and Disease

Cilia are highly conserved organelles that line the cell surface and have specialized functions in the body [7]. These structures are divided into three types: primary, motile, and nodal cilia. 

Primary (or sensory) cilia are non-motile cilia present in a single copy on the surface of most nondividing cells in the body. These structures are key signaling organelles that sense the extracellular environment, serving as chemoreceptors, mechanoreceptors, osmoreceptors, and, in specialized cases, also detect changes in light, temperature, and gravity [7]. Because they mediate development, growth, and repair functions in different organs throughout the body, a diverse group of syndromes collectively termed ciliopathies can result when primary cilia are defective [8]. These syndromes include polycystic kidney disease, nephronophthisis, Bardet-Biedl syndrome, Jeune thoracic dystrophy, Joubert’s syndrome, and Meckel’s syndrome, among others [8].

Motile cilia line the apical surface of epithelial cells along the upper and lower respiratory tract, the male and female reproductive tracts (Fallopian tubes and efferent tubules), and ventricles within the central nervous system [7]. There are typically hundreds of motile cilia per epithelial cell; their primary function is to vectorially propel liquid and mucus parallel to the cell surface in a coordinated fashion. Sperm also have a single flagellum, structurally analogous to the motile cilium, which is responsible for sperm propulsion [9]. 

The multiciliated cells on the airway surface beat in a coordinated pattern to achieve mucociliary clearance, an essential local defense in the upper and lower respiratory tract, moving mucus, trapped inhaled particles, and pathogens. The normal ciliary beat cycle has a strong stroke with the cilia in a straight position, followed by a recovery stroke where motion is initiated from proximal axonemal bending, with little deviation in the longitudinal axis of the cilia [10]. Motile cilia on adjacent cells move synchronously in the same direction. Disruption of airway cilia function may lead to impaired clearance which results in sino-oto-pulmonary infections. Motile cilia dysfunction elsewhere can lead to other features associated with PCD, such as laterality defects, subfertility, and hydrocephalus. 

Nodal cilia (motile monocilia) are expressed briefly during embryological development on the ventral node of the gastrula and consist of nine outer doublets connected by outer dynein arms and inner dynein arms without the central pair, resulting in a “9 + 0” configuration. These organelles have a rotary motion, generating a leftward flow of fluid across the embryonic node, which plays an important role in left-right organ patterning. In the absence of the usual leftward flow, the laterality of organs within the body becomes a random event. 

**Figure 1:**
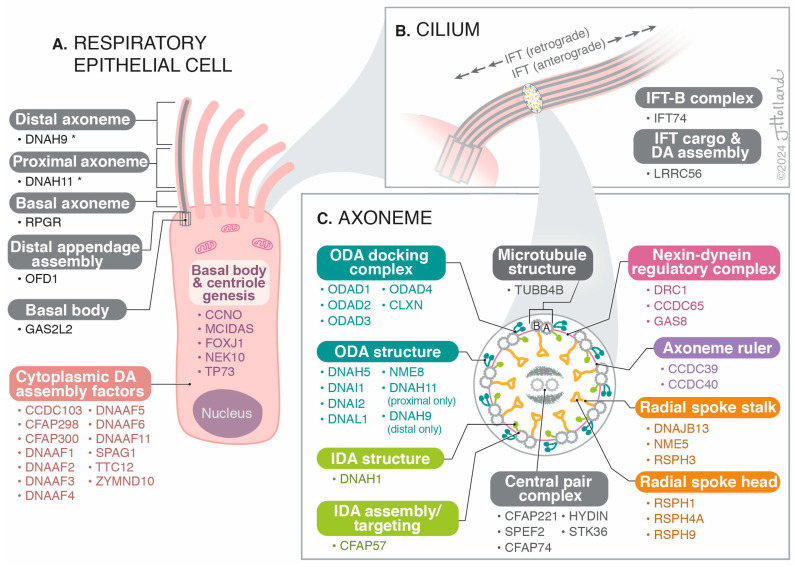
The location and function of the 54 known genes implicated in PCD. (**A**). Respiratory Epithelial Cell. * DNAH9 and DNAH11 are represented twice in this figure, including in panel C, however, these genes are important in ODA structure in different location s along the axoneme length, as demonstrated here. DA = dynein arm. (**B**). Cilium. IFT = intraflagellar transport. (**C**). Axoneme (in cross-section). The outer doublet A and B microtubules are labeled. The cross-section of the axoneme shows the “9 + 2” structure of microtubules in the motile cilium. ODA = outer dynein arm; IDA = inner dynein arm. Illustration by ©Jessica Holland 2024.

All cilia share a core structure of the axoneme (see Figure 1), which contains nine microtubule doublets, each consisting of an A and B tubule, encircling the periphery of the cilia, that is anchored by a basal body in the apical cytoplasm. Motile cilia also have a central pair of microtubules, forming the characteristic “9 + 2” pattern seen in axonemal cross-sections [7,11]. Inner and outer dynein arms extending from the A microtubule interact with the B tubule of the neighboring outer pair, recognizable on electron photomicrographs. The dynein arms are attached to each microtubule doublet at repeated intervals, 24 nm for outer dynein arms and 96 nm for inner dynein arms. The inner dynein arms are linked to radial spokes, which anchor the outer doublets to the central pair [11]. The inner dynein arms and radial spokes interact with the nexin–dynein regulatory complex (N-DRC) that coordinates the activity of the multiple dyneins, regulating motor activity [12]. The radial spokes also regulate dynein arm activity, sending signals from the central apparatus to the dynein arms. The outer dynein arms power ciliary beating in an ATP-dependent fashion, regulated by signals from the central apparatus and the N-DRC, producing a bending that results in a wave-like beat followed by a recovery stroke. All these structures must work in a coordinated fashion and maintain alignment of the doublet microtubules to produce synchronized beating. 

## 3. Clinical Features of PCD

The classic presentation of PCD includes neonatal respiratory distress (NRD), chronic rhinitis, persistent middle ear effusions and recurrent otitis media, and a “wet” cough that occurs daily from infancy. Approximately half of patients have laterality defects including situs inversus [13,14,15]. The characteristic clinical phenotype in PCD includes many common and non-specific symptoms that occur frequently during childhood, resulting in delayed recognition and missed diagnoses.

Evaluating adults for PCD can be equally challenging, as early childhood history (such as details regarding neonatal history) may not be well known by the patient. Virtually every adult with PCD has bronchiectasis, and that finding in combination with a history of chronic or recurrent sinus, ear, and lung infections since childhood should trigger evaluation. Family history is important to assess, though many may not have a family history suggestive of PCD, given the autosomal recessive inheritance pattern for most genes [15]. Adults with PCD may also come to attention during infertility evaluations [9,16]. With the progression of lung function decline, one single-center longitudinal study of 151 adults with PCD reported a 4.6% mortality rate over a median follow-up period of 7 years; the median age of death was 65 years old (range 31–75 years) [17].

### 3.1. Pulmonary Manifestations

With increased recognition and novel diagnostic tests (see Section 4), PCD is increasingly diagnosed in younger children and infants [4]. Early diagnosis with implementation of airway clearance and prompt treatment of infections may be important for long-term health outcomes in patients [17,18].

NRD (defined as supplemental oxygen requirement for more than 24 h when other factors such as meconium aspiration have been excluded) in term infants is reported in about 80% of patients and should raise suspicion for PCD [15]. Daily, year-round, productive (wet) cough that begins in infancy is a distinctive feature of PCD [14]. Despite cough being a compensatory form of clearance of airway secretions in PCD, people with this disease still have frequent episodes of bronchitis and “pneumonia” [19]. Wheezing is relatively uncommon though patients may have rhonchi or crackles that do not consistently clear with cough, and affected individuals can have reduced exertional tolerance [14]. Infectious upper and lower respiratory tract symptoms may temporarily improve (though usually do not completely resolve) with antibiotics [14]. Chest imaging may show bronchiectasis, which is present in roughly 50% of children by 10 years of age and nearly all adults. Pulmonary function testing shows progressive intrathoracic airway obstruction with variable airway hyperreactivity and lung overinflation [20,21,22,23]. 

Lung disease in PCD has long been considered milder than cystic fibrosis (CF); however, children with PCD can have evidence of airflow obstruction at young ages [14,17,18,20,21]. Several studies have now shown that older people with PCD have lower lung function and progressive airway obstruction over time [14,20,21,24]. There is a decline in forced expiratory volume in one second (FEV_1_) and mid-maximal flow rate (FEF_25–75%_) with relative preservation in forced vital capacity (FVC). Some studies have described correlations between increased disease burden on imaging with worse lung function in PCD [17,23,25,26]. Functional changes seem to lag imaging changes [25]. Even when imaging shows disease progression, pulmonary function may not significantly change, suggesting that monitoring spirometry alone may miss disease progression [27]. 

Lung clearance index (LCI) measured by multiple breath washout (MBW) is another measure of lung function. When performing MBW, worse ventilation inhomogeneity is associated with a longer time to equilibrate or clear the inert gas [28]. Recent studies have found that LCI detects abnormal lung function earlier than FEV_1_ or FEF_25–75%_ in children with PCD (similar to what has been reported in CF); in a systematic review, LCI has correlated with imaging findings on high-resolution computed tomography (HRCT) and magnetic resonance imaging (MRI), making it a useful tool for longitudinally monitoring lung disease [29,30]. Work is underway to characterize genotype–phenotype relationships that may elucidate the variation in lung function in PCD, as lung function may be stable for long periods in some patients, but others experience more severe airway obstruction and more rapid decline in lung function. Some may ultimately require lung transplantation [14,17,20,21,24,31]. 

Several factors may influence lung disease severity and progressive decline. Nutritional status has been linked with lung function in several chronic respiratory conditions, including CF, chronic obstructive pulmonary disease (COPD), and non-CF bronchiectasis [32,33,34]. While not well established in PCD [17,24,31,35], some studies have shown lower body mass index (BMI) and lower lung function within certain subgroups and genotypes [20]. Another study demonstrated that women with PCD have lower lung function and a more significant rate of decline than men with PCD [31]. Gender disparities between the development of bronchiectasis in CF as well as in non-CF bronchiectasis (which is more common in post-menopausal women) have similarly been noted and may be related to hormonal fluctuations, but are not well understood [36,37,38].

In the neonatal period, imaging obtained for respiratory distress may show atelectasis, primarily localized to the upper lobes [39]. Other typical findings on chest radiographs include lung hyperinflation, bronchial wall thickening, and laterality abnormalities, including *situs inversus totalis* [40]. Chest computed tomography (CT) scans often show bronchial wall thickening, segmental atelectasis, and with increasing prevalence with age, bronchiectasis [23,40,41]. Bronchiectasis in PCD is most common in the middle and lower lobes, unlike in CF where upper lobe bronchiectasis predominates [23,42]. Bronchiectasis is not specific to PCD, but mucus plugging, tree-in-bud opacities, and atelectasis in conjunction with bronchiectasis are more common in PCD than in other patients with non-CF bronchiectasis due to other etiologies [40,41]. Emphysematous and fibrotic changes are less common in PCD than in other diseases that lead to non-CF bronchiectasis [41]. Gas trapping and consolidation are also noted on CT scans in PCD [40]. Based on these characteristics, a CT scoring system has been developed to identify patients with imaging more suggestive of the PCD diagnosis [43]. This score and familiarity with PCD imaging features may help adult providers consider diagnostic testing for PCD in patients with non-CF bronchiectasis. 

In some studies, higher disease burden on CT has been correlated with worse lung function [23,26] as well as a higher rate of lung function decline in adults and children with PCD [17,25]. In other studies, however, the correlation of CT disease burden with spirometry and LCI has not been seen [44]. Some of these discrepancies may arise from the application of scores that are not specific to PCD to evaluate the severity of imaging findings [45], but the development of Specified PCD Evaluation by CT (SPEC) and the Melbourne-Rotterdam Annotated Grid Morphometric Analysis for PCD (MERAGMA-PCD) may help clarify imaging and lung function correlations in PCD in future studies [46,47,48].

The use of MRI for evaluating ventilation defects and structural lung disease progression has been of interest in PCD. MRI findings in PCD have revealed middle and lower lobe predominance of bronchiectasis, associated with ventilation defects in the upper lobes that may be reflective of mucus plugging preceding structural changes [49,50]. Ventilation defects have been noted on MRI in patients with PCD even with normal spirometry [49,50].

Non-typeable *Haemophilus influenzae*, *Streptococcus pneumoniae*, *Moraxella catarrhalis*, and *Staphylococcus aureus* are the most common pathogens isolated from respiratory cultures in young patients until early adulthood [20,51]. *Pseudomonas aeruginosa* is a more dominant pathogen later in life [13,51]. Prevalence of chronic *P. aeruginosa* infection is estimated to occur in about one-third of patients with PCD [35,51]. Transient infection is more common in children, with only about 5% chronically colonized [27]. Nontuberculous mycobacteria have also been detected in older children and adults [13,17,23,27]. 

The long-term implications of *P. aeruginosa* infection in PCD are unclear. Some studies reported *P. aeruginosa* infection to be associated with lower lung function and increased disease burden on imaging [31,52], while others found no association with FEV_1_. Imaging measures were worse in people with *P. aeruginosa* colonization, though this finding may be related to differences in subject age [51]. Similarly, another longitudinal study showed no impact of *P. aeruginosa* infection on lung function or BMI, though exacerbations were more frequent [35]. 

### 3.2. Sinonasal and Middle Ear Involvement

Sinonasal disease is a hallmark feature of PCD. Daily persistent nasal congestion with rhinitis is present in most patients, beginning in early childhood, and is nearly universal in adults [15,31,53,54]. Symptoms are typically year-round without seasonal variability [15]. These and other symptoms such as facial pain have a significant impact on quality of life but are likely underreported due to their chronic nature from an early age [54,55]. Nearly a quarter have reported a decreased sense of smell, while sleep-disordered breathing (including obstructive sleep apnea) has been noted in nearly half of adults and children [54,55,56]. Symptoms of sleep-disordered breathing are likely underrecognized. A single-center study identified some degree of sleep apnea in all 16 children with PCD evaluated by polysomnography, with a mean apnea-hypopnea index (AHI) of 7.8 [57].

Sinus imaging frequently shows aplasia and hypoplasia of the frontal, sphenoid, and maxillary sinuses, turbinate hypertrophy, septal deviation, mucosal thickening, and sinus opacification [53,58]. Estimates of nasal polyposis vary. Nasal polyps are relatively uncommon in children [54,55], but more common in adults, with a frequency between 15–59% [31,53,54]. Overall nasal polyposis is more common in individuals with PCD than in the general population [54]. Pathogens isolated from the sinuses are typically like the common pathogens in the lower airways [53,55,59].

Endoscopic sinus surgery is performed in roughly 6% of children with PCD, but nearly half of adults have required surgical interventions [31,53]. Endoscopic surgery in patients with PCD was associated with improved quality of life, reduction in infections, and a trend towards improvements in lung function in one single-center study [59]. Another study also described better quality of life after surgery, though olfaction failed to improve [60]. 

Chronic otitis media and middle ear effusions are also common complications, though less specific for PCD in young children [14,15]. Abnormal motile cilia function in the middle ear and Eustachian tube predisposes to recurrent infections [61]. Manifestations in childhood include chronic otitis media. While not specific to PCD, the absence of persistent middle ear effusions reduces the likelihood of PCD [14]. Symptoms include otalgia and often chronic or recurrent otorrhea [62]. Frequent otitis media leads to significant antibiotic exposure in childhood [61], and tympanostomy with pressure equalizing tube insertion is frequently pursued. Many children require repeat procedures [55,61,63]. Pressure equalizing tubes can improve hearing [63,64], but have been associated with chronic complications, including persistent membrane perforations and higher rates of prolonged otorrhea [63,65]. 

Some otologic manifestations of PCD may improve with age, such as recurrent infections, but hearing loss can persist [62]. The importance of regular otolaryngological evaluation was emphasized by one study where 62% of children were found to have hearing loss based on audiograms, but only 38% reported having known hearing loss [55]. Guidelines recommend routine otolaryngological follow-up in children and as needed in adults [66]. Conductive hearing loss has been seen in up to two-thirds of children with PCD [53,63,65], and while it can fluctuate over time, this manifestation can persist into adulthood [62,63,64,67]. Sensorineural hearing loss also occurs, and more commonly persists into adulthood than conductive hearing loss [53,67]. In some studies, a quarter of patients with PCD had undergone speech therapy [61,65], while others required the use of hearing aids [61,62,65]. 

### 3.3. Laterality Defects and Heterotaxy

Approximately 50% of people with PCD have left-right laterality defects due to dysfunction of embryonic nodal cilia, as several genes of motile cilia (in particular those involved in the dynein arms) are essential for nodal cilia development [13,68,69,70,71,72]. Individuals with laterality defects are typically diagnosed with PCD at younger ages than those with typical left-right patterning, or *situs solitus* (SS). Indeed, left-right laterality defects may be appreciated on cardiac and abdominal examinations [14]. 

Most with laterality defects have *situs inversus totalis* (SIT), or complete mirror imaging of thoracic and abdominal organs, with frequencies reported at around 40% in cohorts of patients with PCD [69,70,71]. *Situs ambiguous* (SA) and heterotaxy have been increasingly recognized, with important clinical ramifications. A recent study demonstrated those with PCD and SA have worse pulmonary and nutritional outcomes in childhood compared with those who have SS or SIT [71,73]. Different definitions have been used for heterotaxy, making comparisons between reported frequencies challenging [71]. SA has been identified in approximately 10% of patients with PCD, with around 2–3% having SA associated with complex congenital heart disease [69,70,71]. The overall prevalence of congenital heart disease may be as high as 17% of all patients with PCD (including those with SS), highlighting the importance of a screening echocardiogram in this population [70]. Abdominal ultrasonography in PCD has been recommended to identify potential laterality syndromes such as polysplenia, asplenia, and abdominal *situs inversus* [70].

Certain PCD-related genes that are not involved in the formation or functioning of the nodal cilia (*CCNO*, *MCIDAS*, radial spoke protein genes, central complex genes) have not been associated with laterality defects [70,74,75,76,77]. Conversely, genes not associated with PCD can cause heterotaxy, often as part of different syndromes [78]. 

### 3.4. Infertility and Subfertility

Infertility and subfertility are common, but not universal, findings in men and women with PCD [16,79]. Approximately 80% of men and 60% of women are considered subfertile [16,79]. In men, sperm motility is affected [9], though motile cilia are also present in the efferent ductules of the testes, with recent data suggesting their role is to prevent aggregation of the sperm in the lumen rather than propelling luminal contents forward [79,80,81]. Luminal obstruction due to sperm aggregation has been suggested as another mechanism for infertility in PCD based on animal models [9,80]. Over 20 PCD genes have been associated with male infertility, but the exact mechanism for these individual genes is not well understood [9,82]. 

Although natural conception has been reported in women with PCD, there is evidence of subfertility [16,79,83,84]. Multiciliated cells line the oviducts and assist in gamete transport; therefore, impaired motility is thought to lead to subfertility [16,79,85]. Female fertility has been more difficult to evaluate, as there is no single test to assess fertility related to fallopian tube ciliary motility [16]. The risk of ectopic pregnancy has been a concern, though larger case series have not reported this phenomenon [16,84,86,87]. 

While the degree of respiratory impairment with PCD does not predict fertility, genotype–phenotype relationships have been noted [16]. Despite the similarities between sperm flagella and motile cilia, there are differences in gene expression and composition [9]. Similarly, PCD-related genes with higher expression in testicular cells and fallopian tube epithelial cells are more likely to be implicated in infertility [9,88]. Conversely, there are genes encoding proteins unique to the sperm flagellum that are not seen in the motile cilia of the respiratory tract [9]. These men are infertile but do not have the other characteristic sinopulmonary manifestations of PCD, a condition called multiple morphological abnormalities of the sperm flagella (MMAF) [9,16]. 

Advanced reproductive therapies (ART) have been successful in allowing men and women with PCD to have children despite prior struggles with infertility [79]. Women have been successfully treated with intrauterine insemination (IUI) as well as in vitro fertilization (IVF) [16,79,87]. An increase in respiratory symptoms has been noted during pregnancy for women with PCD [89]. For men with PCD, IVF has been more successful with the use of intracytoplasmic sperm injection (ICSI) with sperm retrieved from the testis versus ejaculated sperm; this may be related to sperm quality [79,90]. Evaluation of men and women for infertility may prompt further testing for PCD in patients who also report chronic respiratory and sinus symptoms or situs abnormalities [79,91].

### 3.5. Hydrocephalus

Though frequently reported in murine models, hydrocephalus is a relatively uncommon finding in humans with PCD [92,93]. Hydrocephalus has been associated with certain PCD genes (*MCIDAS*, *FOXJ1*, *CCNO*, *DNAI2*, *TUBB4B*) with several different proposed mechanisms [76,92,94,95,96,97,98]. The cilia of the ependymal cells lining the ventricles of the brain are thought to help circulate cerebrospinal fluid (CSF); therefore, without functional cilia, this lack of flow has been proposed to contribute to hydrocephalus [92,99]. Diffuse choroid plexus hyperplasia has been noted in patients with *MCIDAS* and hydrocephalus, suggesting that overproduction of CSF by the choroid plexus (perhaps related to ion transport functions of cilia) could also contribute to hydrocephalus in some cases [95,100]. Obstructive hydrocephalus in PCD has also been identified, including aqueductal stenosis requiring surgical intervention with ventriculoperitoneal shunting [96,101]. The flow of CSF by cilia has also been posited to contribute to aqueductal patency [92,100]. Cilia may also play a role in neurodevelopment and neural signaling and, when altered, could contribute to the development of hydrocephalus [92,99]. More research is required to understand the etiology of hydrocephalus in rare cases of PCD, but this finding in a patient with chronic sinopulmonary symptoms or laterality defects should prompt investigation for PCD [96].

## 4. Diagnostic Testing

Currently, there is no gold standard for diagnosis of PCD [102,103]. No single test will identify every person with PCD, nor is there a test that will always exclude PCD. Diagnostic practices vary from country to country related to the cost and availability of expertise [104,105]. Indeed, international society guidelines have differed in their recommended approaches, though they agree that multiple different tests are frequently required to confirm the diagnosis [102,103]. Validated clinical criteria have been proposed to help identify patients with features suggestive of PCD who require further testing, including the 7-point PICADAR (Primary CiliARy DyskinesiA Rule) questionnaire and the 4-item clinical criteria proposed by Leigh et al. [15,93]. The use of clinical criteria may help identify patients most likely to benefit from referral to specialized centers and consideration of additional, more costly specialized testing [93,103,104]. 

Current testing includes nasal nitric oxide (nNO), genetic testing, axonemal ultrastructure analysis using transmission electron microscopy (TEM), immunofluorescent staining, and high-speed video-microscopy analyses (HSVMA), each with benefits but also limitations as discussed below [66,102,103]. Nevertheless, despite the availability of newer diagnostics, there is an ongoing need to refine diagnostic approaches and expand access to testing.

### 4.1. Nasal Nitric Oxide (nNO) Measurements

The observation that people with PCD have significantly lower nNO levels compared to healthy subjects and those with other disease states provided the basis for nNO as an adjunctive test for PCD [13,106,107]. Nitric oxide (NO) is formed by nitric oxide synthases in epithelial cells near the basal bodies of cilia under certain conditions when proper cofactors and substrates (L-arginine and oxygen) are available [108]. In exhaled air, most nNO arises from the sinuses and the upper airways [109]. While NO appears to play a role in ciliary motion, the actual mechanism for low nNO production in PCD is unknown [108].

The basis of nNO measurement uses chemiluminescence to measure emitted light proportional to NO concentration in a sample of gas [110]. Electrochemical analyzers are also available, but they have not been fully tested or validated as a diagnostic tool. In older, compliant subjects, testing is performed during steady, low-flow exhalation with vellum closure to avoid lower airway contamination. Gas is sampled from an inserted nasal catheter in cooperative patients over 5 years of age per American Thoracic Society (ATS) criteria (or over 6 years of age per European Respiratory Society [ERS] criteria) [110,111]. Tidal breathing measures of nNO are useful in younger children unable to perform vellum closure [112]. Currently, normative nNO values are only available for children 5 years and older [102,103]. To account for different flow rates used by different commercially available devices, clinical measures of nNO production are preferably reported as nanoliters per minute (nL/min) rather than parts per billion (ppb) [107]. In chemiluminescent analyzer testing in those at least 5 years of age, an nNO cutoff of <77 nL/min for PCD demonstrates good discrimination against healthy controls, patients with asthma, and COPD [107]. 

Measurements of nNO are predominantly used as an adjunctive tool with reported sensitivity and specificity estimates of 0.90–1.0 and 0.75–0.97 respectively [111]. However, individuals with low nNO levels still require additional testing for diagnostic confirmation. While nNO testing is relatively inexpensive for centers that have the testing capability, the purchase of chemiluminescent analyzers is costly [102]. Moreover, none of these devices are approved by the US Food and Drug Administration for this purpose. Although relatively easy and non-invasive to perform, strict standardized testing procedures should be followed for the most accurate results [110]. 

Non-diagnostic results with nNO above the diagnostic threshold of 77 nL/min have been reported in several genes associated with PCD, including *RSPH1*, *FOXJ1*, *CCNO*, *GAS8*, *CCDC103*, *CFAP221*, *STK36*, *RPGR*, *DNAH9*, *GAS2L2*, *NEK10*, *SPEF2*, *HYDIN*, *TTC12*, *RSPH4A*, and *LRRC56* [74,96,102,113,114,115,116,117,118,119,120,121,122,123,124,125]. Thus, if there is strong clinical suspicion of PCD, additional testing should be performed [103]. Conversely, other conditions with features similar to PCD can lead to low nNO and false-positive screening results, including diffuse panbronchiolitis [126] and CF [13,106,107]. In the North American guidelines, CF should be excluded before evaluation for PCD. People with inborn errors of immunity can also have low nNO levels; this finding coupled with chronic suppurative lung disease from recurrent infections can make it difficult to distinguish those with immunodeficiencies from PCD [127,128]. Transiently decreased nNO may be seen during acute illnesses with viral respiratory infections or bacterial sinus infections [129,130]. Repeat testing on two separate occasions is therefore recommended to confirm reduced nNO levels [102,110].

### 4.2. Ciliary Ultrastructural Analyses Using Transmission Electron Microscopy (TEM)

Ciliary ultrastructure defects in PCD are often seen using TEM. Classification of PCD by ultrastructure defect subtypes has allowed for the characterization of phenotypes [14,20,24,48,103]. First described in 1976, this technique has historically been the mainstay of diagnostic testing for PCD [102,103,131], but 30% of people with PCD have normal axonemal ultrastructure, and alternative diagnostic testing is required in many cases. To perform TEM, sampling of the respiratory epithelium is performed from the inferior nasal turbinate by brush or curette biopsy, or from the lower respiratory tract via brush biopsy during bronchoscopy [103]. The chemically fixed and embedded cells are then thinly sectioned by an ultramicrotome, staining is performed to define the structures of the cilia, and then TEM is used to assess the transverse ciliary structures.

International consensus guidelines for TEM assessment and result interpretation have recently been developed [132]. Class 1 defects are considered classic for PCD, and in conjunction with the clinical features of PCD can confirm the diagnosis [132]. Class 1 defects include outer dynein arm (ODA) defects, outer and inner dynein arm defects (ODA+IDA), and inner dynein arm defects with microtubular (or axonemal) disorganization (IDA+MTD) (Table 1). ODA defects have been identified in 26–59% of patients with PCD, from variants in genes encoding the ODA structural proteins or ODA docking proteins [103,133,134,135]. ODA+IDA defects (associated with variants in genes involved in dynein assembly) have been identified in 6–39% of patients with PCD in studies evaluating TEM [103,111,135]. Finally, IDA+MTD defects most commonly arise from *CCDC39* or *CCDC40* variants [136,137,138,139]. 

In the setting of clinical symptoms of PCD, Class 2 defects can help confirm diagnosis if present across more than one sample and with additional support from other testing, such as genetics. For instance, central complex defects can arise from genetic variants in the radial spoke components of the cilia that normally stabilize the central pair in the middle of the structure (*RSPH4A*, *RSPH1*, *RSPH9*, *DNAJB13*, etc.) [132]. Some sections will show the absence of the central microtubule pair or translocation of an outer microtubule or outer doublet [132]. However, these findings can also be secondary to airway epithelial injury, which can lead to compound cilia, axonemal blebs, and anomalous microtubules [103,132,140]. 

Oligocilia and displacement of the basal bodies into the cytoplasm from where they typically dock at the apical cell surface are consistent with genetic variants that lead to reduced generation of multiple motile cilia, such as *CCNO* and *MCIDAS* [75,76,132]. The few cilia that may be visualized typically have normal ultrastructure, making the diagnosis challenging to distinguish from inadequate sampling. Because IDA abnormalities may be observed in normal subjects in the setting of recent respiratory infections or other epithelial injuries in healthy subjects [103,141], these defects alone are not considered specific for PCD.

The benefits of TEM include high specificity for PCD when classic abnormalities are noted and will identify about 70% of patients with PCD [103]. The sensitivity of TEM is lower, with 30% of patients with PCD having no defects [19,103,135]. Thus, normal TEM alone cannot be used to exclude PCD, and additional diagnostic testing is warranted if strong clinical suspicion remains [102,103]. As noted above, false positives can occur due to acute infections or environmental exposures [102]. Cell culture techniques may help minimize secondary ciliary changes [132,142].

Other challenges with TEM include technique, difficulty acquiring a sample, and result interpretation. The diagnosis of PCD may be missed if there are insufficient numbers of cilia and cells for analysis, especially in cases of PCD with subtle ciliary defects or oligocilia [103]. As many as 40% of biopsies may have inadequate cilia for analysis by TEM [134,143,144]. Moreover, TEM evaluations require considerable experience and expertise, though recent international guidelines for TEM interpretation have helped [1,103]. Additional techniques such as cryotomography or image processing may help further clarify structures for analysis but are still largely experimental [145,146].

### 4.3. High-Speed Video-Microscopy Analysis (HSVMA)

High-speed video-microscopy analysis (HSVMA) is a diagnostic technique using a high-speed digital video camera attached to a microscope to record the movement of cilia on epithelial cells obtained from brush or curettage biopsies of the inferior nasal turbinate or bronchus [103,147]. The frames are then replayed at slower rates to assess ciliary motion patterns and beat frequency; this tool directly assesses ciliary movement and function [103,147]. Cells can be visualized directly for motility defects, or after being grown in tissue culture under air-liquid interface conditions to minimize dysfunction related to secondary ciliary defects [103,142,147]. Results of this testing include qualitative descriptions of the ciliary beat pattern (CBP), ciliary beat frequency (CBF), and measures of particle clearance [10,103,148,149]. Groups have also attempted to use quantitative measures as well as computer programs to analyze the ciliary movement to reduce subjectivity [148,150]. 

HSVMA potentially provides a functional assay for PCD, especially for people without identified genetic or ultrastructural defects [103,151]. Additionally, HSVMA recordings can be reviewed in expert consultation or as part of research [103]. CBP has been correlated with ultrastructure defects and genotypes (Table 1) [10,148,149]. Immotile cilia with slow, short, stiff flickering beat patterns and dyskinetic beat patterns with minimal residual but highly disorganized beating are the most common findings in PCD (typically with ODA or ODA+IDA defects) [10,148,149]. IDA and IDA+MTD defects have been noted to have a stiff forward power stroke with reduced amplitude, though immotile cilia are also seen [148,149]. A rotational beat pattern has sometimes been associated with central complex defects and radial spoke defects [10]. CBF should be incorporated with CBP analysis, as patients with PCD have been found to have increased, decreased, and normal beat frequencies depending on the genotype [10]. HSVMA is not a gold standard for diagnosis, since this approach can still miss individuals with PCD with genotypes that have nondiagnostic, normal, or very subtle changes on HSVMA (e.g., *HYDIN*) [10,114]. Genotypes that cause reduced ciliogenesis (variants in *CCNO*, *MCIDAS*) are also difficult to identify using this method [152]. 

Other limitations to the wider use of HSVMA include the considerable training and expertise required [102,103]. In the hands of experts, HSVMA can have high sensitivity and specificity with a good interobserver agreement for findings consistent with PCD, though the agreement is lower in assessing inconclusive or less classic HSVMA findings [103,151,153]. Additionally, genetic confirmation has not consistently been performed in conjunction with studies evaluating the diagnostic performance of HSVMA [151,153]. There is no standardization of result interpretations despite attempts to reduce subjectivity as previously mentioned, and cell processing and culture techniques can vary at different centers [103]. Inadequate samples and inconclusive results that require repeat sampling are not uncommon, and cell culture techniques have variable rates of success [142,153]. Equipment for testing is also expensive, precluding routine use in resource-limited settings [153]. Thus, while this tool has been incorporated into some diagnostic guidelines, HSVMA is currently available only in select centers in Europe and Canada. Because the technique has not been standardized and validated, it is not recommended by the ATS diagnostic guidelines for PCD [102,154].

### 4.4. Immunofluorescence Microscopy

The use of immunofluorescent staining to localize target proteins within the cilia of respiratory epithelial cells has been used as a research tool for nearly two decades but has more recently been proposed as a diagnostic tool in PCD [102,103,155,156]. It has been used to confirm the absence of proteins integral to ciliary ultrastructure, and several different approaches have been proposed, including panels of multiple antibodies to identify ultrastructure subtypes of PCD based on the pattern of presence or absence of staining [155,157]. These include antibodies to DNAH5 to identify ODA, DNALI1 for IDA, various radial spoke proteins (RSPH1, RSPH4A, RSPH9), and GAS8 to identify the nexin–dynein regulatory complex [136,155,156,157,158,159]. Targeted analysis of genes associated with specific ultrastructure defects can then be undertaken to further confirm the diagnosis [159].

Immunofluorescent staining has demonstrated similar accuracy and sensitivity when compared with TEM [155,157]. Some loss of function and missense variants can be identified [159]. Immunofluorescence analysis is able to be performed on biopsy samples that have fewer ciliated cells compared with the number of cells required for TEM, so immunofluorescence may be able to provide diagnostic information from epithelial biopsies that would otherwise need to be repeated [155]. Processing and analysis using immunofluorescent microscopy may be accomplished more quickly and at a lower cost than TEM [155]. Cost may be further reduced by using a multi-tiered approach with the application of a second panel of different antibodies if no abnormalities are identified after an initial panel of antibodies [155,157,160]. These features of immunofluorescent staining make it an attractive diagnostic tool for lower-resourced settings where other tests may be cost-prohibitive, or expertise is unavailable [155,157]. As additional antibodies to ciliary structural proteins are identified, panels could be expanded in the future to capture more patients with PCD [155,160,161].

Limitations of immunofluorescent staining are similar to TEM, with difficulties identifying patients with PCD with normal ultrastructure, and cannot be used in isolation as a diagnostic tool [155,157]. High-resolution immunofluorescent microscopy approaches in combination with expanded antibody panels mapping to proteins along the length of the cilia and within the cytoplasm have the potential to expand diagnostic accuracy and potentially identify additional gene variants implicated in PCD [161].

### 4.5. Genetics

PCD is a genetically heterogeneous condition, with most of the identified causative genes encoding components of ciliary ultrastructure or proteins involved in the assembly of those components [162]. The majority of the now 54 genes identified to cause PCD (Table 1) demonstrate autosomal recessive inheritance patterns, though genes with autosomal dominant (*FOXJ1*, *TUBB4B*) and X-linked inheritance patterns (*OFD1*, *RPGR*, *DNAAF6*) have been identified [82]. 

Genetic testing can be highly specific for PCD if known pathogenic variants are identified, and the sensitivity of genetic testing continues to improve as newly identified genes are added to commercially available panels [102]. Despite the growing list of causative genes, 20–30% of patients with confirmed PCD based on clinical phenotype and other diagnostic tools do not have any identifiable pathogenic variants in the currently known associated genes [82]. Therefore, negative genetic testing does not exclude PCD. Ongoing efforts to identify additional genes and pathogenic variants in the known genes include the recognition of deep intronic variants and non-canonical splicing variants that may be missed by typical genomic DNA analysis [163,164,165]. The procedure of a simple blood draw is feasible and the cost of testing, including sequencing, has decreased significantly over time [163]. Genetic testing may be relatively unavailable and cost-prohibitive, however, depending on the healthcare system and resource setting [166]. 

Multiple approaches for genetic testing are employed. Several multigene panels are commercially available, which balance capturing the most relevant genes while minimizing costs [82]. Not all panels will contain the same genes, and some include testing of genes for conditions similar to PCD [82]. Genetic panels that include sequence analysis as well as evaluation for large deletions or duplications are recommended [82]. Targeted analysis of either single gene testing in a family where there is a known pathogenic variant, or several genes tailored to a patient’s ethnicity and ancestry are also options [82,167]. Nevertheless, targeted testing and genetic panels will not capture all genetic causes of PCD [168]. If suspicion for PCD remains high after initial negative panel testing, comprehensive genetic testing can include whole exome sequencing or whole genome sequencing [82,163]. Such testing may also identify alternative diagnoses in some cases [163], like inborn errors of immunity, conditions that have clinical features that overlap with PCD [127]. Conversely, identification of two or more variants of uncertain significance in a single PCD-associated gene or in different genes are not sufficient for the diagnosis [82].

Despite limitations, genetic testing has become a first-line test for diagnosing PCD. As genotype–phenotype descriptions emerge, a genetic diagnosis may provide additional prognostic information for patients and providers [14,20,70], and specific genetic mutations may be amenable to precision treatment in the future to restore ciliary function [162]. 

**Table 1:** Summary of diagnostic workup findings based on genes that cause PCD.

Approved Gene Name (Other Gene Names)	TEM ^a^ Defect	nNO ^b^	Ciliary Beat Pattern
***DNAH5***	ODA ^c^	Low	Immotile or stiff
***DNAI1***	ODA	Low	Minimal movement
***DNAI2***	ODA	Low	Minimal movement
***DNAL1***	ODA	Low	Immotile or weak
***NME8*** (*TXNDC3*)	ODA (~66%)	NR ^d^	Normal or immotile
***ODAD1*** (*CCDC114*)	ODA	Low	Immotile or flickering
***ODAD3*** (*CCDC151*)	ODA	Low	Immotile
***ODAD2*** (*ARMC4*)	ODA	Low	Flickering
***ODAD4*** (*TTC25*)	ODA	Low	Immotile or flickering
***DNAH9***	ODA (subtle)	Low or normal	Hypokinetic, reduced distal bend
***CLXN*** (*ODAD5*/*EFCAB1*)	ODA	NR	NR
***CCDC103***	ODA+IDA ^e^, ODA, or normal ^1^	Low or normal	Immotile or normal
***DNAAF1*** (*LRRC50*)	ODA+IDA	NR	Immotile
***DNAAF2*** (*KTU*)	ODA+IDA	Low	Immotile
***DNAAF3***	ODA+IDA	Low	Immotile
***DNAAF11*** (*LRRC6*)	ODA+IDA	Low	Immotile
***DNAAF5*** (*HEATR2*)	ODA+IDA	Low	Minimal movement
***ZYMND10*** (*DNAAF7*)	ODA+IDA	Low	Immotile
***DNAAF4*** (*DYX1C1*)	ODA+IDA	Low	Immotile
***SPAG1*** (*DNAAF13*)	ODA+IDA	Low	Immotile
***DNAAF6*** (*PIH1D3*)	ODA+IDA	Low	Immotile
***CFAP300*** (*C11orf70/DNAAF17*)	ODA+IDA	Low	Immotile
***CFAP298*** (*C21orf59*/*DNAAF16*)	ODA+IDA	Low	Immotile
***CCDC39***	IDA+MTD ^f,2^	Low	Immotile
***CCDC40***	IDA+MTD ^2^	Low	Immotile or stiff
***TTC12***	Subtle IDA+MTD or IDA ^3^	Low or normal	Variable ^4^
***GAS8*** (*DRC4*)	Normal or subtle IDA-MTD	Low or normal	Normal or variable ^4^
***CCNO***	Oligocilia	Low or normal	Inadequate for analysis
***MCIDAS***	Oligocilia	Low	Inadequate for analysis
***FOXJ1***	Oligocilia or normal	Normal	Normal or stiff
***RSPH1***	Central pair complex	Low or normal	Reduced bending angle
***RSPH3***	Central pair complex	Low	Reduced bending angle
***RSPH4A***	Central pair complex	Low or normal ^5^	Rotational pattern
***RSPH9***	Central pair complex	Low or normal ^6^	Rotational pattern
***STK36***	Central pair complex	Normal	Uncoordinated
***DNAJB13***	Central pair complex	Low	Reduced amplitude
***NME5***	Central pair complex	NR	NR
***CFAP74***	Normal	Normal	Rotational, partially stiff
***DNAH1***	Normal	NR	NR
***DNAH11***	Normal	Low	Hyperkinetic
***LRRC56*** (*DNAAF12*)	Normal	Low or normal	Variable ^4^
***IFT74***	Oligocilia, short cilia, MTD	Low	NR
***GAS2L2***	Normal disoriented cilia	Low or normal	Hyperkinetic, normal waveform
***HYDIN***	Normal	Low or normal	Variable ^4^
***CFAP221*** (*PCDP1*)	Normal	Normal	Rotational
***SPEF2***	Normal	Low or normal	Rotational
***DRC1*** (*CCDC164*)	Normal	Low	Hyperkinetic
***CCDC65*** (*DRC2*)	Normal	Low	Hyperkinetic
***NEK10***	Normal, short cilia	Normal	Normal
***TP73***	Oligocilia, short cilia	NR	NR
***OFD1***	Normal	Low or normal	Variable ^4^
***CFAP57*** (*WDR65*)	Normal	Low	Symmetric waveform
***RPGR***	Normal or ODA+IDA ^7^	Low or normal	Variable ^4^
***TUBB4B***	Oligocilia, short bulbous tips	Low	NR

Additional names for the same gene are in parenthesis in column 1. Column 2 lists typical defect(s) seen on TEM for that particular gene. Abbreviations: ^a^ TEM = transmission electron microscopy; ^b^ nNO = nasal nitric oxide, with <77 nL/min abnormal; ^c^ ODA = outer dynein arm; ^d^ NR = not reported; ^e^ IDA = inner dynein arm; ^f^ MTD = microtubular disorganization. ^1^. Hypomorphic variant results in variable EM phenotypes. ^2^. Not all axonemes show microtubular disorganization. ^3^. ODA+IDA TEM defect seen in sperm. ^4^. Defects associated with variable ciliary beat patterns, ranging from immotile, to dysmotile, to near normal. ^5^. Usually low; case with normal nNO in patients with *RSPH4A* variants described in Zhang X et al. [125]. ^6^. Usually low; Yiallouros PK et al. described a case series with 3 of 7 patients with *RSPH9* variants having normal nNO levels [169]. ^7^. Normal is more common. Kuroda A et al. reported a case with a heterozygous *RPGR* variant and ODA+IDA defect on TEM [170].

## 5. Genotype–Phenotype Relationships in PCD

With recognition of an increasing number of genes that impact cilia structure and assembly and the variability in clinical presentations, efforts are underway to characterize specific genotypes associated with different PCD phenotypes. Prior work focused on groupings by ultrastructure changes, however not all genes leading to the same ultrastructure changes have the same clinical manifestations. Indeed, different variants in the same gene resulting in loss of function of a protein versus reduced function of that same protein may have different phenotype ramifications. 

### 5.1. Genotype Relationship with Lung Function

Lower lung function has been seen in patients with variants in *CCDC39* and *CCDC40* in comparison with other genotypes [20,171,172]. These are molecular ruler genes that, when altered, result in IDA+MTD ultrastructure changes. This group of ultrastructure defects that predominantly includes *CCDC39* and *CCDC40* has been found to have a more significant lung function decline over time compared to other ultrastructure groups, which has important prognostic implications [20,172]. In particular, IDA+MTD defects have been shown to have lower lung function based on spirometry and LCI compared with patients who have ODA defects or normal ultrastructure [17,24]. Children with IDA+MTD defects have also been found to have bronchiectasis in more lobes compared with those who have ODA or ODA+IDA defects, as well as more mucus plugging on CT compared to those with ODA defects [14,48]. 

Genes leading to oligocilia (*CCNO*, *MCIDAS*) have been posited to lead to more significant lung disease based on cross-sectional data comparing age and lung function [75,113,173]. These variants are relatively rare within the PCD population, with limited longitudinal data to assess lung function decline over time, and have not been compared with other PCD genotypes. 

Better preserved lung function at the time of diagnosis has been noted in dynein structure gene variants, including *DNAH11* in particular [171]. Patients with variants in *DNAH11* have been noted to have less lung function decline over time [172]. *DNAH11* variants result in normal TEM with altered ciliary beat pattern and function [174]. 

*RSPH1* has also been thought to have better-preserved lung function, in addition to less respiratory distress as discussed below, when compared to age- and sex-matched PCD case controls [74]. The onset of chronic wet cough also occurred later in life [74]. This has been hypothesized to be related to some preserved ciliary function, however initial comparator groups included patients with *CCDC39* and *CCDC40* which may be more severe, and so disease severity related to *RSPH1* is being re-evaluated. 

Interestingly, *DNAH5* (the most common genetic cause of PCD) appears to be phenotypically diverse when topographical analysis is used to evaluate characteristics [171]. Genotype–phenotype relationships for *DNAH5* may be more dependent on the type of variant given the number of different variants in this large gene, with ultrastructure ranging from total absence of ODA (due to variant with premature stop codon) to some ODA still present (in the case of splice variants) [175].

### 5.2. Genotype Relationship with Heterotaxy and Situs Abnormalities

Situs abnormalities have not been seen in association with PCD gene variants that do not have a structural or assembly role in nodal cilia. This includes *CCNO* and *MCIDAS*, which play key roles in cellular differentiation and centriole amplification in multiciliated cells [75,76,113]. Defects in genes encoding proteins absent from nodal cilia, such as radial spoke genes (*RSPH1*, *RSPH4A*, *RSPH9*, *RSPH3*) and central pair genes (*STK36*, *HYDIN*, *DNAJB13*, *CFAP74*), do not cause laterality defects [70,74,117,176,177,178,179]. Laterality defects have not been reported in other rare variants, such as *TTC12*, *GAS2L2*, *CFAP221*, *SPEF2*, *DRC1*, *CCDC65*, *GAS8*, *NEK10*, *NME5*, and *RPGR* [82,124] (Table 2).

Laterality defects and heterotaxy have been described in people with ODA defects (*DNAH5*, *DNAI1*, *ODAD1*, *ODAD3*, *ODAD2*, *CLXN*, *CCDC103*, *DNAH9*), ODA+IDA defects (*DNAAF1*, *DNAAF2*, *DNAAF3*, *DNAAF11*, *DNAAF5*, *ZYMND10*, *DNAAF4*, *SPAG1*, *DNAAF6*, *CFAP300*, *CFAP298*) and IDA+MTD defects (*CCDC39*, *CCDC40*) [82,133]. *DNAH5* variants have had slightly higher rates of laterality defects and heterotaxy than expected, with over 65% of SIT and SA reported in two studies [69,180]. *CCDC103*, another ODA gene, has also been found to have a high frequency of laterality defects [69]. Although acting downstream of *CCNO* and *MCIDAS* in multiciliated cells, *FOXJ1* is required for apical docking of the centrioles in both motile and nodal cilia [76,181] and can result in laterality defects, unlike other genes that cause oligocilia [96,182].

### 5.3. Genotype Relationship with Other Clinical Characteristics

No clear genotype–phenotype relationships have emerged regarding sinus disease, ear infections, and hearing loss, though one group did notice a trend toward increased sinonasal disease in patients with central complex defects [54,171]. *DNAH11* and *RSPH1* have both been noted to have lower frequencies of NRD [74,171]. Conversely, patients with IDA+MTD defects (consisting of patients with *CCDC39* and *CCDC40* variants) were found to have longer neonatal lengths of stays compared to other ultrastructure groups [183].

### 5.4. Genotype Relationship with Subfertility

As previously mentioned (see Section 3), the degree of respiratory disease does not appear to correlate with fertility, but genotype–phenotype relationships have emerged, largely correlated to relative expression and different roles of motile cilia in different locations in the body [9,16,79]. Vanaken et al. found that patients with IDA+MTD as well as ODA+IDA defects were more likely to experience infertility than those with abnormal central complex, ODA alone, or normal TEM [16]. *CCDC39*, *CCDC40*, *DNAAF1*, and *DNAAF11* genes are all highly expressed in the respiratory epithelium, fallopian tubes, and testicular cells [16,79].

*RSPH4A* has low expression levels in the testis and therefore has been thought to affect male fertility less than other variants [16]. A recent description of novel *RSPH4A* variants in unrelated Chinese families, however, noted abnormal sperm morphology; two female patients were also infertile, perhaps related to abnormalities in motile cilia of the fallopian tubes [184]. Although *ODAD1* is expressed in the human testis, it does not appear to be crucial for sperm function and may be compensated by other genes (such as *CCDC36*), so fertility is less affected [88,185]. Conversely, although *DNAH17* is essential for the ODA in the sperm axoneme it is not required for the respiratory cilia axoneme, resulting in isolated male infertility without PCD [186]. Larger international studies of fertility in men and women are required to better understand which genes, when defective, cause subfertility (not necessarily infertility). 

### 5.5. Genotypes with Other Associated Phenotypic Features

Oral-facial-digital syndrome type I is caused by variants in *OFD1* on the X-chromosome, leading to a syndrome of dysmorphic features, low tone, and intellectual dysfunction with some patients also displaying the classic features of PCD such as laterality defects, chronic sinonasal and respiratory tract infections, and NRD [187]. Diagnostic testing has shown low nNO levels with normal ciliary ultrastructure on TEM in these individuals [82,187]. 

Retinitis pigmentosa is a condition with retinal degeneration and progressive loss of vision and can be caused by several different gene variants including *RPGR* which leads to an X-linked inherited form [188]. Patients with *RPGR* variants have rarely also been noted to have recurrent sinus, ear, and lung infections with the development of bronchiectasis but not laterality defects, with *RPGR* playing a role in both respiratory cilia and photoreceptor cilia [188]. TEM may have a normal appearance with abnormal motility on HSMVA, though some ultrastructural defects have also been reported [119,170,188].

There are other syndromes with overlapping features of sensory and motile ciliopathies due to reduced generation of multicilia, like Jeune thoracic dystrophy caused by defects in intraflagellar transport related to *IFT74*, and lissencephaly related to *TP73* defects [189,190]. Another recently identified gene encoding a beta-tubulin isotype, *TUBB4B*, has been linked to three distinct classes of ciliopathic disease, including motile ciliopathy [98].

**Table 2:** PCD-causing genes with associated clinical features reported in the literature.

Gene	Chronic Cough	Bronchiectasis	Chronic Rhinitis	NRD ^a^	Laterality Defects *	Subfertility	Hydrocephalus
***DNAH5***	Y ^b^	Y	Y	Y	Y	Y	NR ^c^
***DNAI1***	Y	Y	Y	Y	Y	Y	NR
***DNAI2***	Y	Y	NR	Y	Y	Y	Y
***DNAL1***	Y	Y	Y	Y	Y	NR	NR
***NME8***	Y	Y	Y	NR	Y	Y	NR
***ODAD1***	Y	Y	Y	Y	Y	N	NR
***ODAD2***	Y	Y	Y	Y	Y	Y	NR
***ODAD3***	Y	Y	Y	NR	Y	Y	NR
***ODAD4***	Y	Y	Y	Y	Y	NR	NR
***DNAH9***	Y	Y	Y	Y	Y	Y	NR
***CLXN***	Y	NR	Y	NR	Y	NR	NR
***CCDC103***	NR	Y	Y	NR	Y	Y	NR
***DNAAF1***	Y	Y	Y	Y	Y	Y	NR
***DNAAF2***	Y	Y	Y	Y	Y	Y	NR
***DNAAF3***	Y	Y	Y	Y	Y	Y	NR
***DNAAF11***	Y	Y	Y	Y	Y	Y	NR
***DNAAF5***	Y	Y	Y	Y	Y	Y	NR
***ZYMND10***	NR	Y	NR	NR	Y	Y	NR
***DNAAF4***	Y	Y	Y	NR	Y	Y	NR
***SPAG1***	NR	Y	Y	Y	Y	Y	NR
***DNAAF6*** ^X^	Y	Y	Y	Y	Y	Y	NR
***CFAP300***	Y	Y	Y	Y	Y	Y	NR
***CFAP298***	NR	Y	Y	Y	Y	NR	NR
***CCDC39***	Y	Y	Y	Y	Y	Y	NR
***CCDC40***	Y	Y	Y	Y	Y	Y	NR
***TTC12***	Y	Y	Y	Y	Y	Y	NR
***GAS8***	Y	NR	NR	NR	NR	Y	NR
***CCNO***	Y	Y	Y	Y	NR	Y	Y
***MCIDAS***	NR	Y	Y	Y	NR	Y	Y
***FOXJ1*** ^AD^	Y	Y	Y	Y	Y	Y	Y
***RSPH1***	Y	Y	Y	Y	NR	Y	NR
***RSPH3***	Y	Y	Y	Y	NR	Y	NR
***RSPH4A***	Y	Y	Y	Y	NR	Y	NR
***RSPH9***	Y	Y	Y	Y	NR	Y	NR
***STK36***	Y	Y	Y	NR	NR	Y	NR
***DNAJB13***	Y	Y	Y	Y	NR	Y	NR
***NME5***	NR	NR	NR	NR	NR	NR	NR
***CFAP74***	NR	Y	Y	NR	NR	Y	NR
***DNAH1***	Y	Y	Y	Y	Y	Y	NR
***DNAH11***	Y	Y	Y	Y	Y	Y	NR
***LRRC56***	Y	Y	Y	Y	Y	Y	NR
***IFT74*** ^+^	Y	Y	Y	Y	NR	Y	NR
***GAS2L2***	Y	Y	Y	Y	NR	NR	NR
***HYDIN***	Y	Y	Y	Y	NR	Y	NR
***CFAP221***	Y	Y	Y	Y	NR	NR	NR
***SPEF2***	Y	Y	Y	Y	NR	Y	NR
***DRC1***	Y	Y	Y	NR	NR	NR	NR
***CCDC65***	Y	Y	Y	Y	NR	NR	NR
***NEK10***	Y	Y	NR	Y	NR	NR	NR
***TP73*** ^+^	Y	Y	Y	Y	NR	NR	NR
***OFD1*** ^X,^^	Y	Y	Y	Y	Y	NR	NR
***CFAP57***	NR	Y	Y	Y	NR	NR	NR
***RPGR*** ^X,^^	Y	Y	Y	NR	NR	NR	NR
***TUBB4B*** ^AD,+^	NR	Y	NR	NR	Y ^?^	NR	Y

All listed genes cause disease by autosomal recessive inheritance unless otherwise specified. Abbreviations: ^a^ NRD = neonatal respiratory distress; ^b^ Y = yes; ^c^ NR = not reported. * Left to right laterality defects include *situs inversus totalis*, *situs inversus abdominalis*, *situs ambiguous*, and heterotaxy. ^?^ Only 1 of 12 cases with *TUBB4B* mutations presented with dextrocardia, none of the *TUBB4B* null mice had any laterality defects, therefore considered questionable [98]. ^X^ Inheritance pattern: X-linked. ^AD^ Inheritance pattern: Autosomal dominant. ^^^ Syndromic motile ciliopathy, *OFDI* characterized by dysmorphic features, hypotonia; *RPGR* characterized by retinitis pigmentosa. ^+^ Overlap between sensory and motile ciliopathy; *TP73* associated with lissencephaly; *IFT74* associated with skeletal dysplasia; *TUBB4B* associated with sensorineural hearing loss.

## 6. Conclusions and Future Directions

PCD is a genetically and phenotypically diverse condition with significant impacts on quality of life due to chronic symptom burden as well as progressive lung function decline. Increased recognition and diagnosis of this rare disease are essential so that patients may be managed and followed at PCD centers with multidisciplinary expertise. Diagnostic evaluations should be considered in adults with non-CF bronchiectasis, chronic upper airway disease, and subfertility, which will provide new insights into the longitudinal outcomes of PCD. 

There is no gold standard to diagnose PCD, nor is there a single test that can reliably exclude the diagnosis. Genetic testing has emerged as a key diagnostic tool and will become more important for diagnosis as more genes and pathogenic variants are identified. Moreover, there are ongoing efforts to refine current diagnostic tests and specifically validate clinical tools for use in PCD, as well as harmonize existing diagnostic guidelines. 

Though there are no PCD-specific approved therapies that restore ciliary function, routine airway clearance and aggressive management of infections are important to preserve lung health and minimize complications [191]. To date, there have only been three randomized controlled trials for PCD. A European study found that people with PCD benefitted from treatment with thrice weekly azithromycin, with decreased pulmonary exacerbation rates and without an apparent increase in antibiotic resistance [192]. More recently, the combination of the selective epithelium sodium channel (ENaC) inhibitor, idrevloride, with hypertonic saline resulted in modest improvements in FEV_1_ after four weeks of therapy, though larger clinical trials are needed to confirm efficacy [193].

Indeed, the creation of specialized PCD networks across North America and Europe has allowed patients to become increasingly involved in clinical trials to evaluate current and novel therapies. Pre-clinical studies with mRNA for correction of variants in *CCDC40* are progressing in Europe, and Phase 1 trials using inhaled mRNA therapy to correct *DNAI1* defects are currently being administered to people with PCD and healthy controls [194]. Other approaches, such as gene editing and read-through therapies, could be on the horizon [194]. 

Better, more sensitive clinical endpoints still need to be defined. The amount of ciliary function restoration required to reduce respiratory symptoms and prevent lung function decline remains to be determined. We expect that a greater understanding of genotype–phenotype relationships and mutation-specific implications will also help us better design clinical trials and tailor treatments to improve outcomes for people with PCD.
